# Evaluating gas chromatography with a halogen-specific detector for the determination of disinfection by-products in drinking water

**DOI:** 10.1007/s11356-018-1419-2

**Published:** 2018-02-28

**Authors:** Anna Andersson, Muhammad Jamshaid Ashiq, Mohammad Shoeb, Susanne Karlsson, David Bastviken, Henrik Kylin

**Affiliations:** 1 Department of Thematic Studies—Environmental Change, Linköping University, SE-581 83 Linköping, Sweden; 2 Present Address: Department of Chemistry, University of Dhaka, Dhaka, Bangladesh; 3 Research Unit: Environmental Sciences and Management, North-West University, Potchefstroom, South Africa

**Keywords:** Drinking water, Disinfection by-products, Trihalomethanes, Haloacetic acids, Haloacetonitriles, Halogen-specific detector

## Abstract

The occurrence of disinfection by-products (DBPs) in drinking water has become an issue of concern during the past decades. The DBPs pose health risks and are suspected to cause various cancer forms, be genotoxic, and have negative developmental effects. The vast chemical diversity of DBPs makes comprehensive monitoring challenging. Only few of the DBPs are regulated and included in analytical protocols. In this study, a method for simultaneous measurement of 20 DBPs from five different structural classes (both regulated and non-regulated) was investigated and further developed for 11 DBPs using solid-phase extraction and gas chromatography coupled with a halogen-specific detector (XSD). The XSD was highly selective towards halogenated DBPs, providing chromatograms with little noise. The method allowed detection down to 0.05 μg L^−1^ and showed promising results for the simultaneous determination of a range of neutral DBP classes. Compounds from two classes of emerging DBPs, more cytotoxic than the “traditional” regulated DBPs, were successfully determined using this method. However, haloacetic acids (HAAs) should be analyzed separately as some HAA methyl esters may degrade giving false positives of trihalomethanes (THMs). The method was tested on real water samples from two municipal waterworks where the target DBP concentrations were found below the regulatory limits of Sweden.

**Electronic supplementary material:**

The online version of this article (10.1007/s11356-018-1419-2) contains supplementary material, which is available to authorized users.

## Introduction

Disinfection to kill harmful pathogens is essential to produce safe drinking water, particularly from surface water sources. Disinfection is often accomplished, by using strong oxidants, such as chlorine, chloramines, chlorine dioxide, or ozone. The chemical disinfectants kill pathogens efficiently, but they also produce unwanted disinfection by-products (DBPs) when reacting with natural organic matter (NOM), anthropogenic contaminants, bromide, or iodide present in the source water (Richardson and Postigo 2015). These DBPs may in themselves be harmful, e.g., having carcinogenic (Cantor 1997; IARC 1995), mutagenic (Cemeli et al. 2006), or genotoxic (IARC 1999) properties. Epidemiological studies suggest increased risk of bladder cancer associated with DBP exposure (Villanueva et al. 2015). Different routes of exposure to DBPs, e.g., drinking, showering, bathing, laundry, and cooking, have been identified, and in one study, the bladder cancer risk was more pronounced by bathing, showering, or swimming in, than drinking the water (Villanueva et al. 2007).

More than 600 DBPs have been identified, but they account for less than 50% of the total organic halogen (TOX) formed (Richardson and Postigo 2015). Among the DBPs, trihalomethanes (THMs) and haloacetic acids (HAAs) have received most attention, and the levels of these DBPs in drinking water are regulated in many countries (Goslan et al. 2009). Most OECD countries have introduced guidelines to control DBPs and minimize consumer’s exposure while maintaining adequate disinfection and control of targeted pathogens. However, these guidelines are based on limited knowledge on the chemical diversity of DBPs. In the USA, THMs, HAAs, and bromate are regulated with maximum contaminant limits (MCL) of 80, 60, and 10 μg L^−1^, respectively (EPA 2010). In the European Union, the total THMs and bromate (BrO_3_^−^) are regulated at 100 and 10 μg L^−1^, respectively (EU 1998). Most previous research has concerned the regulated DBPs, but recently, the interest in unregulated DBPs has increased markedly (Adams et al. 2005; Richardson and Postigo 2015). These include, e.g., haloketones (HKs) and groups of DBPs specifically referred to as emerging DBPs, including haloacetonitriles (HANs), halonitromethanes (HNMs), haloamides, halofuranones, haloacetaldehydes, nitrosamines, halobenzoquinones, iodo-trihalomethanes, and iodo-acids (Richardson and Postigo 2015). These compounds are also formed during disinfection along with THMs and HAAs but typically at lower concentrations (Krasner et al. 2006; Richardson et al. 2007). In spite of the lower concentrations, these unregulated DBPs may represent a larger public health concern, as most of them are more toxic than the regulated DBPs (Bull and Robinson 1986; Plewa et al. 2008; Richardson et al. 2007). In drinking water samples from Spain, France, and the UK, unregulated nitrogen containing DBPs (N-DBPs) accounted for > 90% of the CHO cell cytotoxicity (Plewa et al. 2017); N-DBPs, and haloacetonitriles in particular, represented the forcing agents for cytotoxicity in these water samples. This calls for an adaption of monitoring methods that include DBPs that are of largest public health concern.

During the past three decades, several methods to determine DBPs from different classes have been published, including both GC and LC methods (Chinn et al. 2007; Ding and Zhang 2009; Nikolaou et al. 2002; Pavón et al. 2008; Richardson 2011; Richardson and Ternes 2005; Zhao et al. 2010). A recent review on determination of nitrogenous DBPs concluded that the majority of available methods can determine one or two classes of DBPs only, and called for a development of new methods that can measure several DBP classes simultaneously to improve monitoring (Ding and Chu 2017).

DBPs occur in drinking water at low (ng L^−1^– μg L^−1^) concentrations (Richardson et al. 2007), and the limit of detection (LOD) of the used methods is therefore critical. The LOD depends on both the sample volume and the extraction procedures, among other factors. Differences in physical and chemical properties between different classes of DBPs may make it difficult to extract all target DBPs with a single extraction procedure. There are different methods available for extraction of DBPs. Both liquid-liquid extraction (LLE) (Golfinopoulos and Nikolaou 2005) and solid-phase extraction (SPE) (Buszewski and Szultka 2012; Dittmar et al. 2008; Qian et al. 2015) have been frequently used, but may need fine tuning for specific compound classes. Advantages of SPE over LLE include less solvent consumption, salt free extracts, and that SPE methods can be automated (Buszewski and Szultka 2012).

The volatile DBPs, e.g., THMs, HANs, and HKs, have been quantified using GC-MS (Richardson 2010) or GC with electron capture detection (ECD) (Chinn et al. 2007; Hodgeson et al. 1990; Tominaga and Mídio 2003). For the determination of more polar DBPs with ionizable functional groups (e.g., HAAs), derivatization is necessary prior to separation with GC. The most commonly used derivatizing reagents are diazomethane and acidic methanol (Hodgeson et al. 1995; Sarrión et al. 2000; Xie 2001). The ECD is selective for compounds containing electronegative functions. These include not only halogens but also compounds containing, e.g., nitrogen or sulfur. Further, large amounts of hydrocarbons from the matrix may give rise to negative peaks and noise (Lovelock 1958). Interferences from non-halogenated and co-eluting compounds is therefore a limitation for the analysis of halogenated organic compounds, which is directly associated with the detector.

To address the need of higher selectivity and specificity than given by an ECD, a halogen-specific detector (XSD) was developed that is selective towards halogenated compounds only (OI Analytical 2017). This detector has been used to determine chlorinated fatty acids in biological samples where neither GC-ECD nor GC-MS gave a sufficient chlorine/hydrocarbon selectivity (Nilsson 2004). In the XSD, the GC effluent undergoes oxidative pyrolysis under which halogenated compounds are converted to oxidation products and free halogens. The free halogens react with the alkali-sensitized surface of the cathode, which yields an increased thermionic emission that can be measured (Nilsson et al. 2001; OI Analytical 2017). The XSD has successfully been applied for the analysis of chlorinated compounds such as pesticides (Brown et al. 2011) and halogenated fatty acids (Nilsson et al. 2001; Zhuang et al. 2005).

Within a larger project in which unknown DBPs are identified with ultra-high-resolution spectroscopic methods, we also aim to develop suitable methods for routine monitoring. Given its high selectivity and specificity for halogens, the GC-XSD was expected to give the possibility to detect halogenated DBPs that are not detected with other routine monitoring methods. Here, we present results from a study to investigate the potential of GC-XSD methods for simultaneous monitoring of a range of DBPs of interest to Swedish waterworks, including THMs, HAAs, HANs, HKs, and HNMs.

## Experimental

### Chemicals

LC-MS grade methanol (MeOH) and sulfuric acid 95–97% Merck were acquired from VWR (Spånga, Sweden). Methyl tertiary butyl ether (MTBE) 98%, sodium sulfate (Na_2_SO_4_), and sodium bicarbonate (NaHCO_3_) were acquired from Sigma-Aldrich (Stockholm, Sweden) and ethanol 96% from Solveco (Rosersberg, Sweden). The selected compounds including their class, chemical name, and abbreviation are presented in Table 1.

**Table 1:** Summary of selected compounds along with their class, compound name, and abbreviation

Compound class	Compound name	Abbreviation
Trihalomethanes (THMs)	Tribromomethane (bromoform)	TBM
	Trichloromethane (chloroform)	TCM
	Bromodichloromethane	BDCM
	Dibromochloromethane	DBCM
Haloacetonitriles (HANs)	Bromochloroacetonitrile	BCAN
	Dibromoacetonitrile	DBAN
	Dichloroacetonitrile	DCAN
	Trichloroacetonitrile	TCAN
Haloketones (HKs)	1,1-Dichloro-2-propanone	DCP
	1,1,1-Trichloro-2-propanone	TCP
Halonitromethanes (HNMs)	Trichloronitromethane (chloropicrin)	TCNM
Haloacetic acids (HAAs)	Monochloroacetic acid	MCAA
	Monobromoacetic acid	MBAA
	Dichloroacetic acid	DCAA
	Dibromoacetic acid	DBAA
	Trichloroacetic acid	TCAA
	Bromochloroacetic acid	BCAA
	Bromodichloroacetic acid	BDCAA
	Chlorodibromoacetic acid	CDBAA
	Tribromoacetic acid	TBAA

### Standard solutions

Standards of THMs (bromoform, chloroform, bromodichloromethane, dibromochloromethane) and other neutral DBPs, viz. HANs (dichloroacetonitrile, dibromoacetonitrile, bromochloroacetonitrile, trichloroacetonitrile), HKs (1,1-dichloro-2-propanone and 1,1,1-trichloro-2-propanone) and a HNM (trichloronitromethane), and HAAs (monochloroacetic acid, monobromoacetic acid, dichloroacetic acid, dibromoacetic acid, trichloroacetic acid, bromochloroacetic acid, bromodichloroacetic acid, chlorodibromoacetic acid and tribromoacetic acid) were from Restek, acquired from Teknolab Sorbent (Kungsbacka, Sweden). Additional standards of 1,2-dibromopropane (97%) and 1-chlorodecane were acquired from Sigma-Aldrich (Stockholm, Sweden).

Stock solutions in methanol containing 0.2 μg μL^−1^ (stock solution 1, Table S1), 0.02 μg μL^−1^ (stock solution 2), and 0.002 μg μL^−1^ (stock solution 3) were prepared for every THM, HAN, HK, HNM, and HAA (see Table S1 in supplementary information for details). The stock solutions were added to samples of Milli-Q water for calibration, as well as to MtBE for direct GC-XSD determination. The surrogate standard 1,2-dibromopropane was prepared in methanol and the recovery standard 1-chlorodecane was prepared in MTBE.

### SPE procedure

SPE was performed with Bond Elute PPL (modified styrene divinylbenzene polymer, 200 mg in 3-mL cartridges, Agilent Technologies, acquired from Scantec, Partille, Sweden) using a vacuum manifold (10 port, Sorbent, Göteborg, Sweden).

#### Neutral DBPs (THMs, HANs, HKs, HNMs)

To test the method capacity to detect the selected target DBPs, Milli-Q water (1 L) was spiked with standard mixes to a concentration of 20 μg L^−1^ of each compound and the pH was lowered to ≈ 2 with sulfuric acid (1 mol L^−1^). The surrogate standard 1,2-dibromopropane (50 μg, i.e., 25 μL of stock solution 1) was added before extraction. The SPE cartridges were activated by passing MeOH (2 × 3 mL) through the cartridge followed by acidified Milli-Q water (3 mL, pH ≈ 2). The cartridges were placed on a manifold. The water samples and the cartridges were connected via PTFE tubes (ID 2 mm) with one end attached to the cartridge via an adaptor and the other end inserted in the glass bottles containing the water samples. The glass bottles were placed 1.5 m above the cartridges for the extraction. The water samples were fed to the cartridges by gravity at a flow rate of not more than 10 mL min^−1^. After extraction, vacuum was gently applied for 30 s to remove excess water. MeOH (100 μL) was added to the cartridges, after which the analytes were eluted with MtBE (2 mL) and the extracts were collected in 4-mL glass vials. Fifty microgram recovery standard (10 μL stock 1 in Table S1; 1-chlorodecane) was added to each extract, and 1 mL was transferred to auto sampler GC vials for GC-XSD analysis (see Chart S1 in supplementary information for a schematic overview of the method). The remaining extracts were stored at − 20 °C.

#### Haloacetic acids

The pH of Milli-Q water (50 mL) was adjusted to ≈ 0.5 with sulfuric acid (1 mol L^−1^) and spiked with 20 μg L^−1^ HAA mix standard solution (100 μL of the HAA mix standard stock solution 1, see Table S1). SPE was performed as described above after which the analytes were eluted with MeOH (1 mL) and MtBE (2 mL). EPA method 552.3 (Domino et al. 2003) was used for esterification. Briefly, the extracts were transferred to 15-mL glass tubes and acidic methanol (2 mL) was added. To initiate the methylation reaction the glass tubes, with Teflon-lined screw caps were placed in a water bath (50 ± 2 °C, 2 h ± 10 min). After methylation, sodium sulfate solution (5 mL, 150 g L^−1^) was added followed by vortexing, after which the test tube was left standing until the phases were clearly separated. The upper phase, containing the esters, was transferred to a 10-mL test tube. Saturated sodium bicarbonate solution (1 mL) was added to raise the pH of the acidic extracts to a neutral pH. After vortexing again, the tubes were left for phase separation, and the upper layers containing esters were transferred to 4-mL glass storage vials. Of the extracts, 1 mL aliquots were transferred to GC vials for GC-XSD analysis (see Chart S2 in supplementary information for a schematic overview of the method). The remaining extracts were stored at − 20 °C.

### Survey of waterworks and drinking water

After the above tests, the described method for neutral DBPs was used to determine DBPs in real drinking water samples collected from two waterworks, Berggården in Linköping municipality and Borg in Norrköping municipality, Sweden. Samples (1 L) were collected at three different sites before and three sites after disinfection. The waterworks use the same surface water source, Motala ström, Berggården, approximately 50 km upstream of Borg, but different disinfection systems (Figs. S1 and S2). Briefly, Berggården used UV followed by hypochlorite for disinfection. At Borg, disinfection was performed using chloramination, a supposedly milder disinfectant. For further details, see the supplementary information.

### Instruments

Gas chromatography was performed on an Agilent 6890 interfaced to a 5973 mass spectrometer (MS) (Agilent Technologies, Avondale, PA, USA). The GC was also equipped with a detector system with a photoionization detector (PID) followed in tandem by an XSD (OI Analytical, College Station, TX, USA). The PID was not used for this work. Consequently, the PID will only be mentioned below when relevant for the discussion. Separation took place on a DB-5 column (30 m × 0.25 mm i.d. × 0.25-μm film thickness, (J&W Scientific Sacramento, Folsom, CA, USA), with the flow split 1:9 between the MS and the PID-XSD. The MS was operated with electron ionization at 70 eV under full scan mode (*m/z* 40–550). The GC inlet was operated in splitless mode with the oven temperature permitting solvent trapping of the analytes at the head of the column. The GC temperature program was 27 °C held isothermally for 1.3 min, 7 °C min^−1^ to 80 °C followed by 30 °C min^−1^ to 250 °C, held isothermally for 5 min. PID sweep flow and XSD air flow were 30 and 30 mL min^−1^, respectively. Helium was used as carrier gas. The optimized conditions gave a good separation of the chromatographic peaks for the identification of the neutral DBPs. The analytes were identified from the retention times of the individual analytes established using the MS detector and by comparison of the mass spectral data of pure compounds with the NIST 2005 database and specific diagnostic ion fragments of each component.

### Quality assurance and control

Standard operation procedures were adopted, following a strict method protocol ensuring consistency in method execution. Analytes were identified by comparing the retention time (± 2%) with the corresponding standards. The surrogate standard (1,2-dibromopropane) was added to water samples and consistently used to calculate the recovery for extraction quality control. The recovery standard (1-chlorodecane) was added to the final extracts and was used for quantification of each analyte taking into account differences in chromatographic runs and extracted volumes. Calibration curves (4–6 points) were constructed in a concentration range described in Table 2, depending on the individual compound, see supplementary materials Tables S2–S4 for peak area data for target DBPs, surrogate standard, and recovery standard for both calibrations and water analysis.

**Table 2:** Retention times for studied neutral DBPs at the optimized temperature program, calibration range for each compound, correlation coefficients for calibration curves, extraction recoveries with standard deviations, and estimated limits of quantifications (LOQ)

Compound	Retention time (min)	Calibration range (μg L^−1^)	Correlation coefficient (*R*^2^)	Mean recovery (%)	Standard deviation	Estimated LOQ (μg L^−1^)
Chloroform	3.2	0.2–20 0.2–5	0.9963 0.9992	53	0.024	0.2
Trichloroacetonitrile	3.7	0.05–1	0.9996	65	0.103	0.05
Bromodichloromethane	4.2	0.2–5	0.9982	64	0.033	0.05
Dichloroacetonitrile	4.5	0.05–1	0.9991	45	0.008	0.05
1,1-Dichloro-2-propanone	4.6	0.05–1	0.9999	44	0.016	0.05
Trichloronitromethane	5.5	0.05–1	1.0000	69	0.104	0.05
Dibromochloromethane	5.9	0.05–5	0.9986	72	0.032	0.05
Bromochloroacetonitrile	6.5	0.05–1	0.999	71	0.017	0.05
1,1,1-Trichloro-2-propanone	6.8	0.05–0.5	0.9998	84	0.035	0.05
Bromoform	8.0	0.05–0.5	0.9975	81	0.032	0.05
Dibromoacetontrile	8.7	0.05–1	0.9973	23	0.004	0.05

**Fig. 1:**
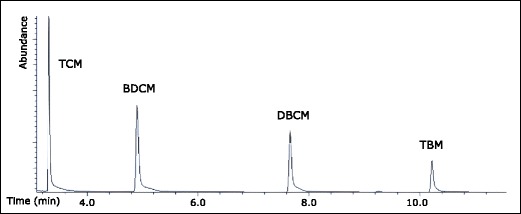
GC-XSD chromatogram of THMs (40 μg L^−1^) in Milli-Q water

**Fig. 2:**
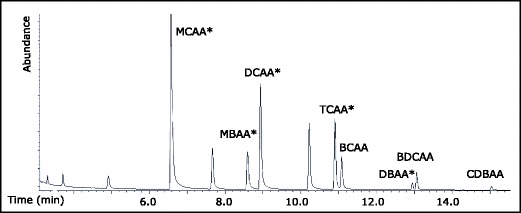
GC-XSD chromatogram of an HAA standard (10 ng μL^−1^ of each) in MtBE. TBAA is not visible here as it was almost entirely converted to TBM. The HAAs marked with asterisks are regulated in many countries

**Fig. 3:**
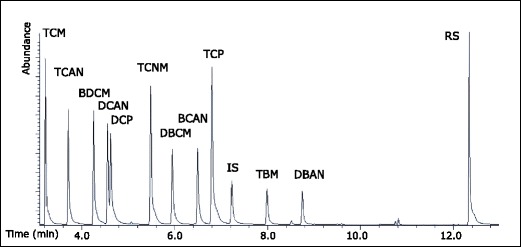
GC-XSD chromatogram of neutral DBPs (20 μg L^−1^ of each) in Milli-Q water. The concentration of the internal standard (1,2-dibromopropane) was 50 μg L^−1^. The recovery standard (1-chlorodecane, 50 μg) was added to the sample vial just prior to injection

**Fig. 4:**
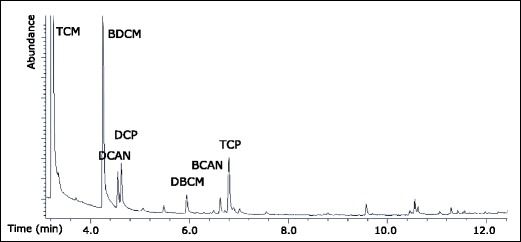
GC-XSD chromatogram of DBPs in tap water from Berggården

The equipment was rinsed with methanol, and laboratory blanks were analyzed repeatedly to assess potential sample contamination. The extraction recoveries for each target DBP were determined by performing three extractions of Milli-Q water (1 L) spiked to 10 μg L^−1^ of each target DBPs. Because of the low noise of the detector, the limiting factor for this instrumental setup was the tailing of each peak (Figs. 1, 2, 3, and 4). Therefore, the limit of quantification (LOQ) was set equal to the concentration of the lowest standard concentration giving peaks that could be integrated. No further attempt was made to calculate the limit of detection (LOD) of individual analytes. GC-XSD data was collected in MSD Chemstation D.03.00 and exported to Excel 2013 for further processing**.**

## Results and discussion

### Method performance

#### Haloacetic acids

Swedish work health regulations will not allow the use of diazomethane for routine laboratories. We, therefore, chose a derivatization method based on acidic methanol. The methyl esters of all the HAAs were detected with the XSD (Fig. 2), but the methylation efficiency differed between individual compounds affecting the overall recovery. In Fig. 2, the five HAAs (HAA5) that are regulated in some countries are marked with an asterisk (*), and among these, DBAA had the lowest response. It has been shown that methylation of the more sterically hindered HAAs, including TBAA, CDBAA, and BDCAA, are not complete even after 2 h of reaction with acidic methanol (Domino et al. 2003). Longer derivatization time did not enhance the derivatization efficiency. In addition to HAAs, peaks with retention times corresponding to THMs for the initial temperature program appeared in the chromatogram (Fig. 2). The MTBE blanks showed no presence of THMs, but HAA methyl esters may degrade into the corresponding THMs during gas chromatography (Heller-Grossman et al. 1993). The peak that appeared at retention time around 10.2 (Fig. 2) coincides with TBM indicating transformation of TBAA. The identity of TBM was also confirmed with GC-MS. Since unstable methyl esters were degraded to THMs giving false positive quantifications of THMs, simultaneous determination of THMs and HAAs should be avoided. In the further work, the method was optimized and evaluated for the measurements of neutral DBPs only.

#### Neutral DBPs

The neutral DBPs were well separated except for DCAN and DCP which co-eluted (Fig. 3). The temperature program was adjusted to optimize separation between DCAN and DCP, but they could not be fully separated in this GC system. The retention times, calibration ranges, correlation coefficients, extraction recoveries with standard deviations, and LOQs for each target DBP are shown in Table 2. The average extraction recovery was 63% with lower recoveries of analytes containing two halogen atoms than analytes containing three halogen atoms. The LOQ determined for all the neutral DBPs was 0.05 μg L^−1^ except for chloroform that was 0.2 μg L^−1^. The LOQ for chloroform was higher than the other analytes, as trace levels of chloroform were present in the Milli-Q water blanks. Even though the detector has lower response for bromine than for chlorine, bromoform and dibromoacetonitrile were clearly detected at spiking concentrations of 0.05 μg L^−1^.

The linearity was tested by plotting the signal ratio of the analyte response and the recovery standard response on *Y*-axis versus concentration on *X*-axis. The correlation coefficients (*R*^2^) were above 0.99 for all target DBPs.

These calibration curves differed for different DBP compounds, as the detector response depends on the number of chlorine and bromine atoms in the compound. The calibration curves also varied due to systematic differences in extraction efficiency among compounds. Hence, one single surrogate standard cannot represent the extraction behavior of all the analytes studied. For this reason, the analyte to surrogate standard signal ratio was not used for quantification. The surrogate standard was instead used to control the extraction performance when analyzing real water samples.

The LOQ can be further decreased by extracting larger volumes or by optimizing the extraction conditions. Further, additional classes of toxicologically important DBPs can likely be determined simultaneously, given that they have similar characteristics in terms of extractability and volatility. One example of such a class is haloamides that might contribute to a major part of the DBP toxicity (Plewa et al. 2017; Wagner and Plewa 2017).

### XSD performance

The XSD is highly selective for halogenated compounds, more so than the commonly used ECD (Nilsson et al. 2001). The response varies between different halogens and is higher for chlorine than for bromine. The high selectivity combined with the virtual absence of noise gave very clean chromatograms (Figs. 1, 2, 3, and 4), the stable baseline allowing detection of low concentrations. The start-up time of the detector to get a stable baseline was 30 min. In this study, we focused on known DBPs of relevance to Swedish waterworks, but the high selectivity for halogenated compounds should also render the XSD useful for feedback between routine and research analysis. In other words, the XSD might be used as a tool to discover halogenated compounds that can be further investigated and identified with other methods.

The downside of this instrument setup was peak tailing in the XSD chromatograms. The tailing of the XSD peaks was observed for all compounds studied, but was absent in the MS chromatograms. Even when bypassing the splitter between the MS and PID-XSD tailing remained. Consequently, the tailing in our experiments was likely induced by the PID-XSD setup or the XSD itself. Testing a GC-XSD setup without a PID would be useful for further evaluation.

### Analysis of drinking water samples

DBP concentrations were determined at different stages of the water treatment process in two waterworks, three before and three after the point of chlorination or chloramination (Table 3). The recoveries of the surrogate standard were within the acceptable range 70–130% for all samples at both waterworks. At Berggården, 7 out of the 11 neutral target DBPs were found and quantified in the water samples after chlorination. TCAN, TCNM, TBM, and DBAN were below the LOQ. The dominant DBPs at Berggården were TCM and BDCM with average concentrations of 8.1 and 1.6 μg L^−1^, respectively. The average total THM (sum of TCM, TBM, BDCM, and CDBM) were 9.9 μg L^−1^. Some DBPs were found at levels around 0.2 μg L^−1^ including DCAN, DCP, DBCM, and TCP. On the other hand, the dominant DBPs formed during chloramination at Borg were TCM and DCP, with average levels of 0.4 and 0.3 μg L^−1^, respectively. DCAN was also detected at Borg, while the other target DBPs were below LOQ (Table 3). The total concentrations of THMs at Berggården and Borg were well under the limit 100 μg L^−1^ set by the Swedish Food Administration (Livsmedelsverket 2015).

**Table 3:** Concentrations of DBPs in drinking water samples taken from the waterworks Berggården (Linköping) and Borg (Norrköping). The results cover target DBP concentrations at six different steps in the water purification process, three before and three after disinfection using sodium hypochlorite (NaOCl) at Berggården and monochloramine (NH_2_Cl) at Borg

	Berggården						Borg					
Compound	Raw water (μg L^−1^)	Sand filtration (μg L^−1^)	UV treatment (μg L^−1^)	Chlorination NaOCl (μg L^−1^)	Finished water (μg L^−1^)	Tap water (μg L^−1^)	Raw water (μg L^−1^)	Carbon filtration (μg L^−1^)	Sand filtration (μg L^−1^)	Chloramination NH_2_Cl (μg L^−1^)	Finished water (μg L^−1^)	Tap water (μg L^−1^)
Chloroform	< LOQ	< LOQ	< LOQ	7.1	8.2	9.0	< LOQ	< LOQ	< LOQ	0.3	0.4	0.4
Trichloroacetonitrile	< LOQ	< LOQ	< LOQ	< LOQ	< LOQ	< LOQ	< LOQ	< LOQ	< LOQ	< LOQ	< LOQ	< LOQ
Bromodichloromethane	< LOQ	< LOQ	< LOQ	1.6	1.5	1.8	< LOQ	< LOQ	< LOQ	< LOQ	< LOQ	< LOQ
Dichloroacetonitrile	< LOQ	< LOQ	< LOQ	0.3	0.3	0.3	< LOQ	< LOQ	< LOQ	0.07	0.08	0.09
1,1-Dichloro-2-propanone	< LOQ	< LOQ	< LOQ	0.3	0.2	0.2	< LOQ	< LOQ	< LOQ	0.3	0.3	0.4
Trichloronitromethane	< LOQ	< LOQ	< LOQ	< LOQ	< LOQ	< LOQ	< LOQ	< LOQ	< LOQ	< LOQ	< LOQ	< LOQ
Dibromochloromethane	< LOQ	< LOQ	< LOQ	0.1	0.2	0.2	< LOQ	< LOQ	< LOQ	< LOQ	< LOQ	< LOQ
Bromochloroacetonitrile	< LOQ	< LOQ	< LOQ	0.07	0.07	< LOQ	< LOQ	< LOQ	< LOQ	< LOQ	< LOQ	< LOQ
1,1,1-Trichloro-2-propanone	< LOQ	< LOQ	< LOQ	0.2	0.2	0.2	< LOQ	< LOQ	< LOQ	< LOQ	< LOQ	< LOQ
Bromoform	< LOQ	< LOQ	< LOQ	< LOQ	< LOQ	< LOQ	< LOQ	< LOQ	< LOQ	< LOQ	< LOQ	< LOQ
Dibromoacetontrile	< LOQ	< LOQ	< LOQ	< LOQ	< LOQ	< LOQ	< LOQ	< LOQ	< LOQ	< LOQ	< LOQ	< LOQ

*< LOQ* below limit of quantification

A broad spectrum of neutral halogenated DBPs were successfully extracted from and detected in real-life samples using SPE coupled with GC-XSD. Figure 4 shows a GC-XSD chromatogram of a tap water sample after distribution from Berggården. This demonstrates the selectivity of the XSD, which produces clean chromatograms even of real drinking water samples and allows successful determination not only of regulated DBPs but also of toxicologically relevant nitrogen containing DBPs not yet regulated.

## Conclusions

With climate change, increasing population and decreasing access to clean water supplies, relevant control of DBPs will likely be an increasing public health concern. The work presented here is part of a larger project within which we aim to map DBP formation with ultra-high-resolution spectroscopic methods. Such methods will, however, not be possible to use for routine monitoring at waterworks. Part of the necessary vigilance for the future will have to be access to analytical methods that allow cheap and reliable determination of key DBPs. Our choice to test the XSD for this purpose was based on previous experience of its selectivity for halogens which enabled the analysis of chlorinated fatty acids where neither GC-ECD nor GC-MS gave sufficient selectivity or specificity for real samples. The GC-XSD setup is easy to operate and likely gives sufficiently high selectivity and specificity for routine DBP monitoring, but might also find its role in DBP research, where unknown halogenated compounds can be picked out with the XSD and further identified with other methods. Hence, the XSD may be used for routine monitoring, but it might also become a tool to identify future problematic DBPs.

## Electronic supplementary material

ESM 1 (PDF 142 kb)
