# Supplementary material for: Evaluating gas chromatography with a halogen-specific detector for the determination of disinfection by-products in drinking water
Source: Environ Sci Pollut Res Int. 2018 Feb 28;26(8):7305–14. doi: 10.1007/s11356-018-1419-2 (PMC6447507; doi:10.1007/s11356-018-1419-2)
Supplement: Supplementary file 1 — (PDF 142 kb) [file 11356_2018_1419_MOESM1_ESM.pdf]

Evaluating gas chromatography with a halogen specific detector for the determination of disinfection by-products in drinking water

Supplementary information

Anna Andersson[a], Muhammad Jamshaid Ashiq[a], Mohammad Shoeb[a†] , Susanne Karlsson[a], David Bastviken[a], Henrik Kylin[a, b]*

[a] Department of Thematic Studies – Environmental Change, Linköping University, SE-581 83 Linköping, Sweden

[b] Research Unit: Environmental Sciences and Management, North-West University, Potchefstroom, South Africa

[†] Permanent address: Department of Chemistry, University of Dhaka, Dhaka, Bangladesh

Table S1 List of stock standard solutions

Standard	Stock 1 (µg µL^{-1})	Volume (mL)	Stock 2 (µg µL^{-1})	Volume (mL)	Stock 3 (µg µL^{-1})	Volume (mL)
THM-mix	0.2	10	0.02	5	0.002	5
DBP-mix	0.2	10	0.02	5	0.002	5
HAA-mix	0.2	5	-	-	-	-
1,2-dibromopropane	2	10	0.2	5	-	-
1-chlorodecane	5	10	-	-	-	-

Treatment process at Berggården waterworks, Linköping

The treatment capacity at Berggården treatment plant is 30 000-40 000 m^3 day^{-1} and the water work serves around 110 000 customers in Linköping. At Berggården, water from the Motala Ström river is pumped through a 14 km pipe to the plant. Firstly, leaves, algae and larger particles are removed by macro filtration (mesh 0.03 mm). Then the water passes through 8 parallel rapid sand filters, to further separate solid particles. The rapid sand filters are 50 m^2 and have a bed depth of 1 m and operates at 8-10 m h^{-1}.

The next treatment step is slow sand filtration. There are eight parallel filter beds and these beds are much larger, 1000 m^2, and the water passes through the 1 m deep sand filter in approximately eight hours (flow rate ~ 0.17-0.24 m h^{-1}). During slow sand filtration the water is also treated biologically; microorganisms in the sand filters remove organic matter from the water.

After slow sand filtration the water is disinfected by UV. There are eight UV units, operating at 254 nm and each unit has 50 W m^{-2} intensity and flow rate 380 m^3 h^{-1}. After UV treatment the pH is adjusted with lime to avoid corrosion of the distribution pipes, and NaOCl is added to prevent bacterial growth in the distribution system. The processed water is transferred to a storage reservoir (11 000 m^3) before distribution to consumers. The full treatment process, from raw water to finished drinking water takes about 24 hours.

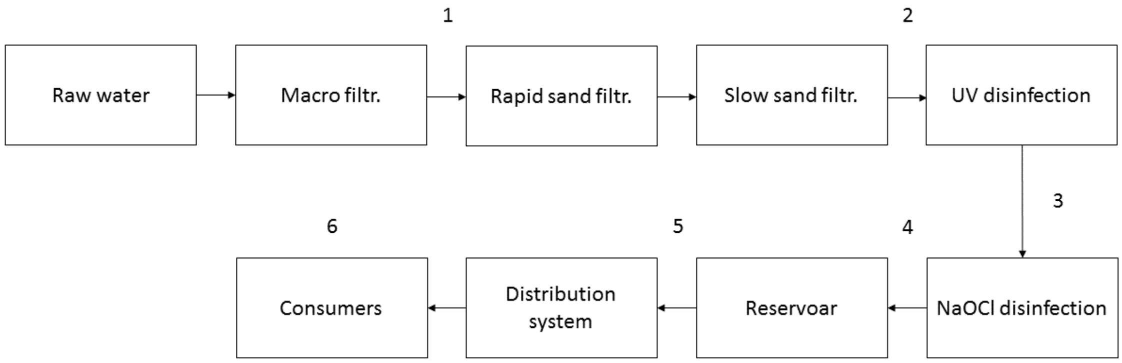

Fig. S1 Treatment processes at Berggården waterworks, Linköping. The sampling points are indicated 1-6.

Treatment process at Borg waterworks, Norrköping

Water is taken from the Motala Ström river, downstream Lake Glan and Linköping. Borg services 115 000 customers and has a production capacity of approximately 47 500 m^3 day^{-1}. First, carbonate is added to raise alkalinity. Aluminum sulfate is added as coagulant and flocculate tiny dispersed particles and some dissolved solutes in the water. There are six parallel flocculation chambers. After flocculation, flocs settle in sedimentation tanks. Then fast carbon filtration, reduce some of the organic chemicals as well as taste and odor producing compounds. The carbon filters also catches flocs that did not settled as sediment. There are 12 parallel carbon filters and the flow rate is ~ 4 m h^{-1}. Lime is then added to raise pH from 6.2-6.4 to 7.0-7.2 creating an environment suitable for the microbes in the sand filters. As the water passes through the sand filters, particles of foreign matter are trapped in the matrix and dissolved organic material is metabolized by the bacteria, fungi and protozoa growing on the surface of the sand. There are eight filter chambers with a total area of 5300 m^2 and the bed depth vary between 0.5 to 1.3 meters (flow rate ~ 0.27 m h^{-1}).

After slow sand filtration, lime and ammonium sulfate is added followed by sodium hypochlorite. In this step, monochloramine is formed in the water stream. Hypochlorite is added slightly in access to enable a minor primary disinfection effect. The pH is raised to 8.3-8.7 to favor the formation of monochloramine over other possible chloramines and to prevent corrosion in the distribution network. After disinfection, the water is transferred into a reservoir (5500 m^3) from where it is distributed to the consumers. Monochloramine has less direct disinfection effect, but persists longer than compound than hypochlorite and prevents bacterial growth in the distribution system.

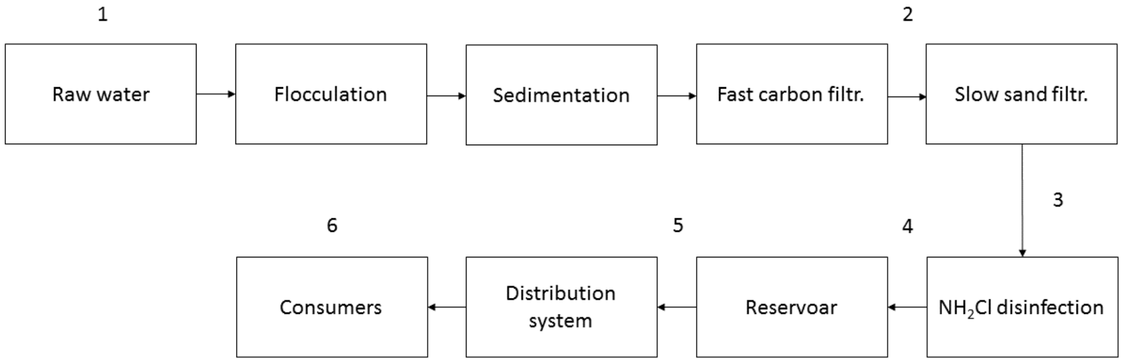

Fig. S2 Treatment process at Borg waterworks, Norrköping. The sampling points are indicated 1-6.

(HAAs).

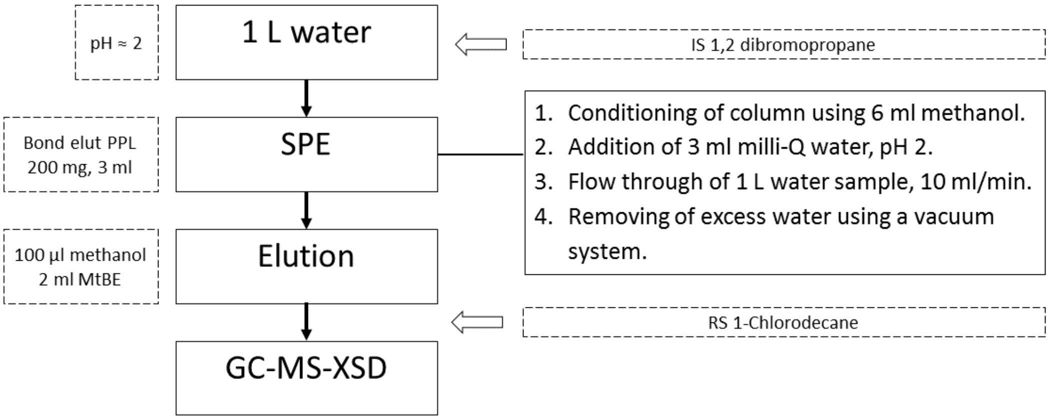

Chart S1 Determination of neutral halogenated drinking water disinfection by-products.

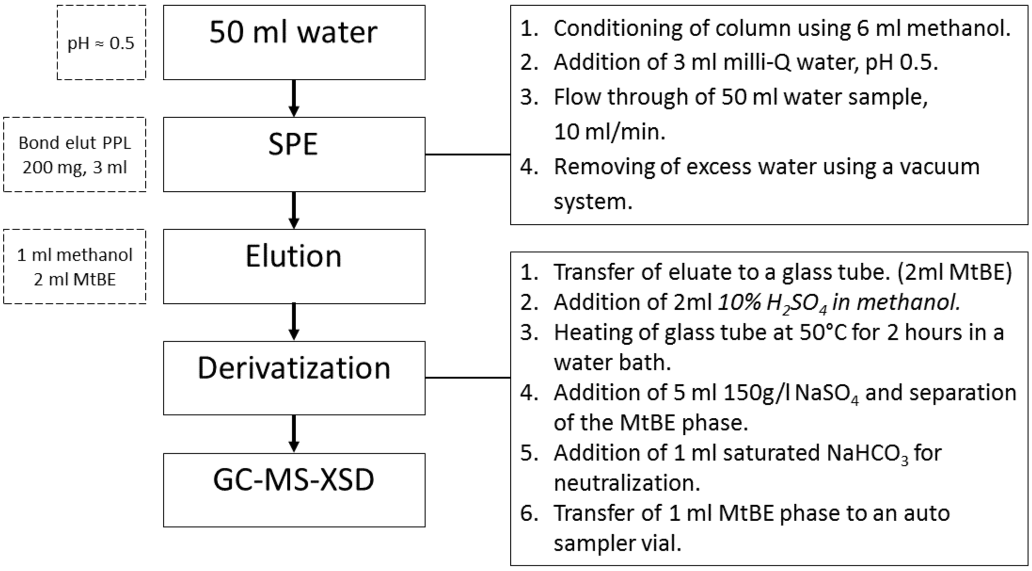

Chart S2 Determination of acidic halogenated drinking water disinfection by-products (HAAs).

Table S2 Peak areas for each target DBP together with recovery standard (RS) and internal standard (IS).

Conc (μg/l)	TCM	TCAN	BDCM	DCAN	DCP	TCNM	DBCM	BCAN	TCP	TBM	DBAN	Area RS	Area IS
0.05	NA	35758	NA	39200	40367	77747	33422	26004	72841	15010	12694	23244157	NA
0.1	NA	67828	NA	85064	89889	151345	79072	66559	170907	33477	33477	21756758	NA
0.2	934464	NA	251582	168553	185570	NA	112459	127499	354738	NA	NA	22226282	NA
0.5	1559776	581927	739329	412210	513850	881129	414779	340091	983749	144694	125416	23455279	8556035
1	2315220	1142068	1355767	762199	1015456	1719456	830816	703378	2013127	335011	253715	22720815	6938949
5	7069370	3552600	5458404	3155237	4143007	5410345	3561969	3016792	7758249	1507394	1231071	22629111	6384246
10	9152225	6190850	7911666	6053521	7739294	9563088	5790622	5921812	14687552	2809230	2513013	22003933	9337827
20	15782515	12742056	14345430	9925042	13669649	19310138	10871546	10430543	24616816	5385412	4641501	21974783	7247022

Table S3 Peak areas for target DBPs, recovery standard (RS) and internal standard (IS) for the water samples from Berggården.

Sample (μg/l)	TCM	TCAN	BDCM	DCAN	DCP	TCNM	DBCM	BCAN	TCP	TBM	DBAN	Area RS	Area IS
Raw water	25062	-	2151	-	-	-	-	-	-	-	-	28868556	8785722
Sand filtration	67562	5385	8302	-	-	10230	4543	-	-	-	-	20018875	9597161
UV treatment	62041	-	3568	-	-	-	-	-	-	-	-	21333430	9858232
NaOCl Chlorination	5404348	6234	1670224	240495	292029	44038	89492	35513	367386	-	-	20769718	9500679
Finished water	7780740	-	1970844	280935	264130	49029	164347	42744	479674	-	-	26196126	10790010
Tap water	6952682	-	1945983	199764	194511	22693	160201	23996	281243	-	-	21411237	10180401

Table S4 Peak areas for target DBPs, recovery standard (RS) and internal standard (IS) for the water samples from Borg.

Sample (µg/l)	TCM	TCAN	BDCM	DCAN	DCP	TCNM	DBCM	BCAN	TCP	TBM	DBAN	Area RS	Area IS
Raw water	25216	-	-	-	-	-	-	-	-	-	-	24942926	6725360
Carbon filtration	59143	3338	8175	-	-	5462	2056	-	-	-	-	22855933	9540318
Sand filtration	53845	-	-	-	-	-	-	-	-	-	-	22283235	9418658
NH_2Cl Chlorination	446972	2501	70695	67745	281622	6497	4249	16708	59323	-	-	22144749	8988406
Finished water	524557	-	65662	68361	303251	2305	1909	15450	62728	-	-	21709901	9582330
Tap water	583407	-	72591	78159	401654	-	-	14174	43732	-	-	21487871	9448603
